# Deep Learning-Based Glaucoma Screening Using Regional RNFL Thickness in Fundus Photography

**DOI:** 10.3390/diagnostics12112894

**Published:** 2022-11-21

**Authors:** Hyunmo Yang, Yujin Ahn, Sanzhar Askaruly, Joon S. You, Sang Woo Kim, Woonggyu Jung

**Affiliations:** 1 Department of Biomedical Engineering, Ulsan National Institute of Science and Technology (UNIST), Ulsan 44919, Republic of Korea; 2 Department of Chemical and Biomolecular Engineering, University of Illinois at Urbana-Champaign, Champaign, IL 61820, USA; 3 Incipian LLC, Laguna Niguel, CA 92677, USA; 4 Department of Ophthalmology, Ulsan University Hospital, University of Ulsan College of Medicine, Ulsan 44033, Republic of Korea

**Keywords:** glaucoma, normal-tension glaucoma, color fundus photographs, optical coherence tomography, retinal nerve fiber layer, convolutional neural networks, screening

## Abstract

Since glaucoma is a progressive and irreversible optic neuropathy, accurate screening and/or early diagnosis is critical in preventing permanent vision loss. Recently, optical coherence tomography (OCT) has become an accurate diagnostic tool to observe and extract the thickness of the retinal nerve fiber layer (RNFL), which closely reflects the nerve damage caused by glaucoma. However, OCT is less accessible than fundus photography due to higher cost and expertise required for operation. Though widely used, fundus photography is effective for early glaucoma detection only when used by experts with extensive training. Here, we introduce a deep learning-based approach to predict the RNFL thickness around optic disc regions in fundus photography for glaucoma screening. The proposed deep learning model is based on a convolutional neural network (CNN) and utilizes images taken with fundus photography and with RNFL thickness measured with OCT for model training and validation. Using a dataset acquired from normal tension glaucoma (NTG) patients, the trained model can estimate RNFL thicknesses in 12 optic disc regions from fundus photos. Using intuitive thickness labels to identify localized damage of the optic nerve head and then estimating regional RNFL thicknesses from fundus images, we determine that screening for glaucoma could achieve 92% sensitivity and 86.9% specificity. Receiver operating characteristic (ROC) analysis results for specificity of 80% demonstrate that use of the localized mean over superior and inferior regions reaches 90.7% sensitivity, whereas 71.2% sensitivity is reached using the global RNFL thicknesses for specificity at 80%. This demonstrates that the new approach of using regional RNFL thicknesses in fundus images holds good promise as a potential screening technique for early stage of glaucoma.

## 1. Introduction

Glaucoma is a chronic and irreversible disease caused by damage to the optic nerve that can lead to vision loss and blindness [1,2,3]. Glaucoma is typically characterized by the progressive degeneration of retinal ganglion cells, resulting in morphological changes in both the optic nerve and retinal nerve fiber layer (RNFL) [4,5,6,7,8]. As no optimal cure for glaucoma currently exists, typical treatments only aim to interrupt the progression of functional damage and visual impairment. Thus, early detection of glaucoma is critical to prevent significant vision loss.

Retinal fundus photography monitors the retinal structure and is also widely used for initial glaucoma screening. Fundus photography provides the two-dimensional surface of the retina, enabling the identification of the characteristic changes in the optic disc due to increased intraocular pressure. These changes include rim thinning, notching, cupping, and altered cup-to-disc ratio [4,5,6,7,8]. However, it suffers from low reproducibility and sensitivity and is inherently limited regarding quantitative and volumetric deformations, such as thinning of RNFL [9,10]. In addition, grading glaucoma based on fundus photographs may vary depending on the physician’s experience and subjective factors. Thus, current fundus photography technique, though still a valuable tool in glaucoma care, may not provide a general screening capability in primary care settings.

To date, optical coherence tomography (OCT) has become an indispensable tool to detect glaucoma, providing cross-sectional tissue structures noninvasively in real time. Since its introduction, OCT has been widely used in ophthalmology, given its unique ability to image deep ocular tissues and quantitatively analyze the RNFL [11,12,13]. Currently, OCT allows rapid, three-dimensional, wide-angle imaging of retinal structures and has become a standard tool in clinical practice. Numerous studies have also demonstrated that OCT is an effective tool for glaucoma diagnosis, progression monitoring and quantification of structural damage [14,15,16,17,18]. 

However, despite its increasing distribution, routine checkups using OCT remain highly limited in most countries. In addition, using OCT for screening purposes or monitoring glaucoma patients is difficult because of the expensive costs and the rarity of trained experts outside specialized clinics. It is estimated that up to 90% of glaucoma patients do not know they have the disease in developing countries [19]. Therefore, there is a pressing need for a cost-effective strategy for glaucoma screening in low-resource settings.

With the recent development of deep learning (DL) techniques, several researchers have shown the capability of automated ocular disease screening using color fundus photography by training DL models [20,21,22,23,24,25,26,27,28]. At the initial development of glaucoma screening DL models, researchers trained the model using human-labeled fundus images [29]. However, human labeling has limited potential due to subjective labeling and low reproducibility [30,31,32]. These obstacles were overcome by hiring OCT data as the reference for objective assessment of a given fundus photo with OCT’s quantitative structural information [33]. Moreover, studies showed that trained DL models could infer quantitative values of glaucomatous structural deformation from fundus photographs, for example, retinal nerve fiber thinning and neuro-retinal rim loss [33,34].

Nonetheless, practical challenges remaining for the DL algorithms for screening with the purpose of early prevention. Despite secured objective assessment of glaucoma by OCT measurement as a reference, high accuracy and sensitive screening for early-stage glaucoma could not be guaranteed exclusively from overall or summarized quantitative values. This is largely because localized defects of RNFL are not well reflected in averaged thicknesses. It had been shown that the screening test based on the average of RNFL was poor in detecting early stage glaucoma while, antithetically, using regional RNFL thinning showed high screening sensitivity [14]. Therefore, to develop a highly accurate and sensitive screening protocol for detecting glaucoma at an early stage, quantitative regional information should be used to reference assessment.

In this study, we acquired the data from normal tension glaucoma (NTG) patients with the intention to develop a screening protocol for early-stage glaucoma. The NTG is the subcategory of primary open angled glaucoma (POAG) with intraocular pressure (IOP) within the normal range and is observed more frequently in the Asian population [3,35,36]. NTG has similar symptoms to other glaucoma types, such as glaucomatous optic neuropathy, and corresponding visual field (VF) defects [37]. We focused on the localized RNFL defect which is commonly observed both in high IOP POAG and NTG [38,39,40]. With the dataset from NTG patients, therefore, an inclusive glaucoma screening protocol using RNFL thickness can be tested. 

We developed a DL model that infers regional RNFL thickness values from color fundus photos. The proposed model is designed to infer regional RNFL thickness from each sectionalized optic disc image along 12 subregions. Predicted RNFL thickness is categorized into three thinning levels by comparison with the normative reference and utilized for a highly sensitive screening protocol for glaucoma detection.

## 2. Materials and Methods

### 2.1. Data Preparation

We acquired the data from 303 patients diagnosed with normal tension glaucoma (NTG) in Ulsan University Hospital ophthalmic clinic to build our DL model and its validation. The enrolled NTG patients had a peak intraocular pressure of consistently ≤21 mmHg without glaucoma medication, a normal open angle, typical glaucomatous optic nerve and visual field changes, and the absence of an ocular or systemic disorder responsible for the optic nerve damage. Before the diagnosis, at least two reliable and reproducible visual field examinations were obtained using the Humphrey field analyzer and the Swedish Interactive Threshold Algorithm 24-2 test program (HFA 24-2; Carl Zeiss Meditec, Inc., Dublin, CA, USA). A total of 557 eyes from 303 patients were acquired for the dataset. The average of the mean deviation (MD) of collected eyes is −4.69 dB and about 72.6% of cases were in early stage (MD > −6 dB). The averaged visual field index (VFI) of collected eyes is 88.39%. The dataset comprised color fundus photographs (TRC-NW8, Topcon, Tokyo, Japan) and spectral-domain OCT (SD-OCT) scans (Triton and Atlantis, Topcon, Tokyo, Japan) from collected eyes.

Collected raw data were preprocessed for preparing the training set, as follows. The color fundus photographs of size 3216 × 2136 were processed in four steps to compartmentalize the optic disc region: (1) convert color fundus image to gray scaled image, (2) perform the Gaussian filtering with kernel size of 65 × 65, (3) brightest spot identification by scanning the max intensity pixel, since the center of the optic disc is generally the brightest in fundus photos, and (4) cropping a circular region of 320 × 320 pixels around the center of the optic disc. The cropped fundus photographs were manually reviewed by an expert to ensure the centering of the optic disc. Manually filtered preprocessed images were sorted into an image dataset without artifacts such as whitening, blurring, dusk, camera artifacts, dust and darkness. OCT measurements were acquired from the report generated by analysis software (IMAGEnet6, version 1.24, Topcon, Japan). From a given SD-OCT scan, the software averages data over 360° for the global RNFL thickness and 30° for corresponding regional RNFL thicknesses along the circle with a diameter of 3.45 mm centered at the optic disc. We assumed that a preprocessed fundus photograph and corresponding OCT scan were rotationally aligned. A total of 940 pairs of color fundus photographs and SD-OCT scans within 180 days (mean 31.4 days and standard deviation 62.9 days) were collected. Fundus photographs were sectionalized along 30° to produce a fan-shaped optic disc image toward each sub-regional direction and matched with corresponding RNFL thickness, accounting for 11,280 data pairs. A summary of collected raw data is given in Table 1.

Collected raw data was split into training (80%) and test (20%) sets. The random sampling process was at the patient level to avoid biased estimation of test performance. For the training process, data from 242 patients with 730 pairs for global RNFL and 8760 pairs for segmented were used. The remaining 210 pairs for global RNFL and 2520 pairs segmented from 61 patients were utilized as the holdout test set for model performance evaluation and further screening protocol test.

In addition, we acquired 583 fundus photographs from 522 normal patients who visited the Ulsan University Hospital HealthCare Center. Among the fundus photographs from normal patients 103 suspicious fundus photos from 93 patients were selected by a glaucoma expert; however, OCT measurements were not performed for normal patients. Additional fundus images from normal patients were used to verify the applicability of our approach. This study for clinical use was approved by the Ethics Committee of Ulsan University Hospital (Approval No. 2020-09-001).

### 2.2. CNN Architecture for RNFL Prediction and Training

**Figure 1:**
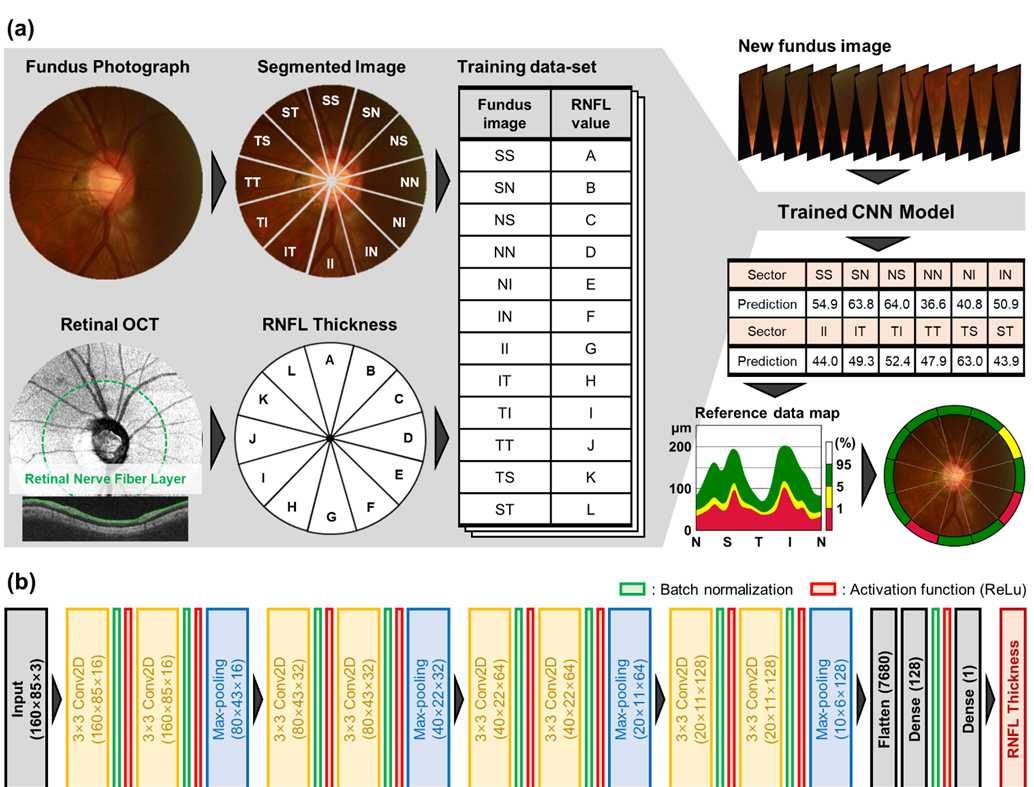
Overview of the proposed study. (**a**) Fundus images and OCT images were segmented into 12 subsections and then the segmented images were paired with the RNFL thickness measurements from OCT to train the model. The trained CNN model can predict RNFL thickness from a subdivided retinal image. The label of the thinning level for an estimated RNFL of a given region is determined by comparison with the normative reference data. (**b**) The details of CNN architecture for RNFL thickness estimation from segmented retinal fundus images are presented. Note that the model for estimation of global RNFL has the input size as 320 × 320 × 3 and thus sizes of the following convolution blocks were adjusted.

To predict RNFL thickness from fundus photographs, we utilized a convolutional neural network (CNN) [41]. Figure 1b shows that the proposed model architecture consists of four convolution blocks and fully connected layers. Each convolution block comprises double convolutional layers with a kernel size of 3 × 3 and a stride of 2. After the convolutional layers, batch normalization and max-pooling layers are used for stable and fast learning [42]. The depth of the convolutional layers increases from 16 to 128 to extract enough image features for accurate estimation of RNFL thickness. The fully connected layer with 128 nodes is placed after the final convolution block, which gathers high dimensional features extracted by convolution blocks. The final node returns the estimated RNFL thickness from the merged and weighted features that transform into a numeric value from the input fundus photograph. Nonlinearity is introduced by the rectified linear unit (ReLU) activation at each convolution and fully connected layer [43]. At each step of the training, randomly sampled 32 pairs have formed a minibatch. To increase the diversity and heterogeneity of the training set, the data of the fundus photographs were augmented by applying random contrast, brightness, hue and saturation. The mean squared error (MSE) is used for the loss function and model parameters were updated by the Adam optimizer [44]. 

We additionally trained the model with an identical method to predict global RNFL thicknesses from a whole optic disc image for comparison with former studies. The proposed CNN models were trained and validated based on the five-fold cross-validation method using the training set from 242 patients. In total, 5 models were trained for each global and regional RNFL prediction, and the final models were then selected based on performance evaluation using validation sets.

Training, validation, image processing, and visualization were performed using the Python (version 3.6.12) programming language with open-source libraries, TensorFlow (version 2.4.1), OpenCV (version 4.5.1) and Matplotlib (version 3.3.4). 

### 2.3. Estimation and Categorization of RNFL Thickness for Glaucoma Screening

Two trained CNN models predicted the RNFL thickness value from the whole and sectioned optic disc images. For a given fundus image input, CNN outputs RNFL thickness in μm scale. For each eye, we predicted 13 RNFL values: the global RNFL (360 degrees) and 12 regional RNFL corresponding to each direction (30 degrees).

The thinning level of RNFL is categorized into three levels (green, yellow, and red) by comparison between OCT measured and the normative database [45]. The green indicates normal range, between 95% to 5% of the reference data; the yellow indicates borderline range, between 5% to 1% of the reference data; and red indicates beyond range, outside 1% of the normative reference data [46].

We used the predicted thinning level of RNFL for screening criteria, as determined by a comparison between the CNN predicted RNFL thickness and the normative database. In a previous study, the screening capability of RNFL measurement using SD-OCT was investigated [14]. Positive diagnosis was made for glaucoma if any abnormal or borderline level is included in interested regions of a given fundus image. Comparison of various combinations of subregion were tested to demonstrate that screening performance strongly depends on subregion variations.

**Figure 2:**
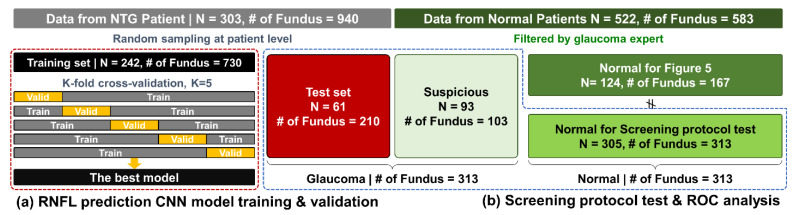
Summary of research workflow. (**a**) Data acquired from NTG patients were utilized for model training and evaluation. (**b**) Screening test and ROC analysis performed with test set plus data from normal patients.

Receiver operating characteristic (ROC) analyses were performed for global and regional predicted RNFL thicknesses. The ROC curves reveal the tradeoff between the true positive rate (TPR or sensitivity) and the false positive rate (FPR or 1-specificity). The area under ROC curve (AUC), sensitivity for fixed specificities at 80% and 95% and corresponding threshold of RNFL thickness values were investigated. The research workflow and how acquired datasets were used at each step is summarized in Figure 2.

## 3. Results

### 3.1. Model Evaluation and Regional RNFL Thinning Level 

**Figure 3:**
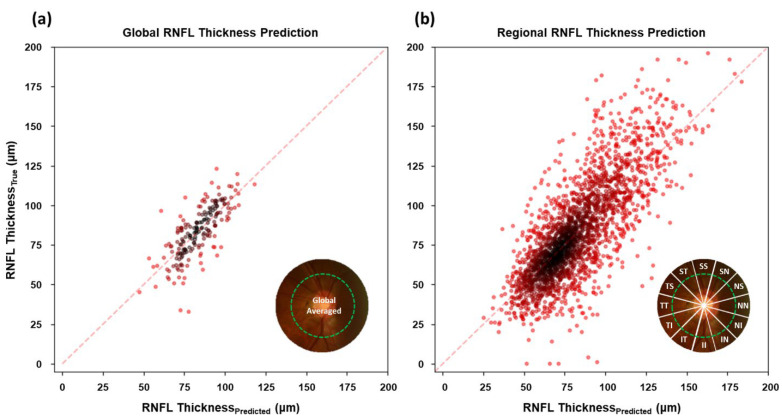
Scatterplots representing the relationship between predicted RNFL by trained CNN from optic disc photographs and actual OCT-measurements. (**a**) The results of global RNFL estimation from the whole optic disc image; (**b**) the results of regional RNFL estimation from the parted optic disc images along 12 directions. Data is based on the test set.

Figure 3 shows the relationship between estimated RNFL thickness from optic disc photograph by trained CNNs and OCT measurements in the test set for the global and the regional RNFL. To verify the model performance, we confirmed the mean absolute error (MAE) between predicted and OCT measured for the regional RNFL prediction from the trained model, the R-squared value and the Pearson’s correlation coefficient, as shown in Table 2.

The MAE for the global RNFL prediction from the proposed architecture achieved 9.38 μm, which is a similar error range compared to a previous study [33], which was 7.39 μm. The MAE for the regional RNFL thickness prediction is more significant than the global RNFL prediction. The higher MAE in regional RNFL prediction is attributable to the reduced information amount due to a sectionalized optic disc image for input to the DL model compared to the non-sectionalized image.

The predicted values strongly correlate with the ground truths, as shown in Figure 3 by the linear trend along a wide range of RNFL thicknesses in both regional and global averages. Correlation between predicted and OCT measured RNFL confirms that the suggested CNN architecture was trained as desired and properly working for RNFL thickness estimation from the optic disc image. A summarized comparison between averaged OCT measurement and trained CNN prediction on RNFL in the test dataset along regions is given in Table 3.

**Figure 4:**
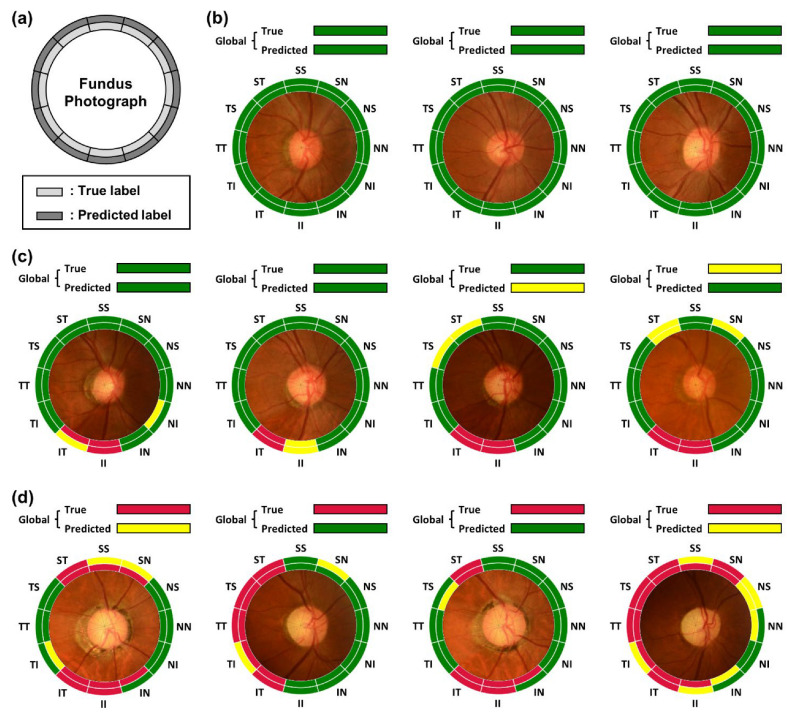
Evaluation of the labeling results of RNFL thinning level with representative examples. (**a**) Representation format. The inner color label indicates the true thinning level from OCT measurement and the outer color label is the predicted level from CNN estimation. (**b**) All normal, (**c**) partially damaged, and (**d**) severe cases. Data is based on the test set.

In Figure 4, representative examples of prediction from the test set are shown for various RNFL thinning progression. Figure 4a shows an example with true and predicted RNFL thinning level for each sub-region. Examples in Figure 4b are all regions in normal: Figure 4c globally normal but with partially damaged RNFL, and Figure 4d for severe cases in which progression of RNFL thinning is acute.

In the example, the prediction of the RNFL thinning level is not always correct since there is an error in the prediction of RNFL thickness. However, the inference capability of the regional RNFL thinning levels from a fundus photograph could be useful for screening glaucoma when OCT measurement is not available. It is also helpful to distinguish the condition briefly among different levels of progression in RNFL defects.

### 3.2. Regional RNFL Thinning and Glaucoma Screening

**Figure 5:**
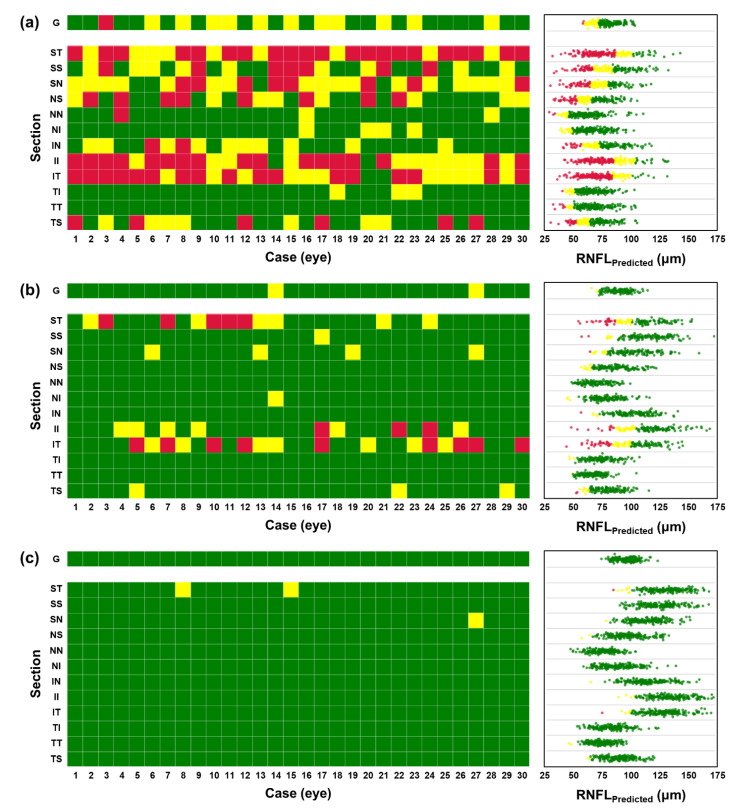
Prediction and categorization of RNFL for different patient groups; (**a**) glaucoma (N = 61, 210 cases), (**b**) suspicious (N = 93, 103 cases), and (**c**) healthy (N = 124, 167 cases) patients. The colored tiles showing the categorization of RNFL thinning level from a randomly selected 30 eyes and scatters on the right side represent all estimated RNFL values in (μm) with categorized color. Each row indicates regions from the global (G) and 12 regional subsections (from ST to TS).

We applied our prediction protocol to three groups to reveal different RNFL thinning levels among distinct patient groups. In Figure 5, prediction results are shown for 30 randomly selected cases for each of the following groups of patients: (a) glaucoma (NTG), (b) suspicious, and (c) normal patients. The colored grid represents the prediction of the RNFL thinning level using trained CNN for the global and 12 regional parts. The scatters indicate predicted RNFL values with corresponding colors of thinning level. From the results, we have confirmed that the categorization of RNFL thinning level based exclusively on the global can often lead to false negative outcomes.

Shown in Figure 5a,b, are the categorization results of averaged RNFL from many glaucomatous eyes and most suspicious eyes in the normal range. However, a clear distinction is disclosed in the categorized regional RNFL thinning level. Notably, directional thinning of RNFL at the superior and inferior region is clearly shown from glaucomatous eyes. More specifically, directional thinning is predicted in suspicious patients from superior-temporal (ST), inferior (II), and inferior-temporal (IT) regions. Regional thinning of RNFL around the optic disc was previously reported, significantly thinning of the superior and inferior region strongly correlated in the early stage of glaucoma progression, and much quicker to become thinner than other regions [47,48,49]. Our results from estimated RNFL thickness from fundus photograph using trained CNN showed a similar tendency compared to previous findings. Therefore, prediction of RNFL thinning level along 12 regions from optic disc image with trained CNN could be useful for screening glaucomatous patients with OCT-based RNFL measurements. A summary of estimated RNFL from three groups as the average and the standard deviation for the global and 12 regions is given in Table 4.

**Figure 6:**
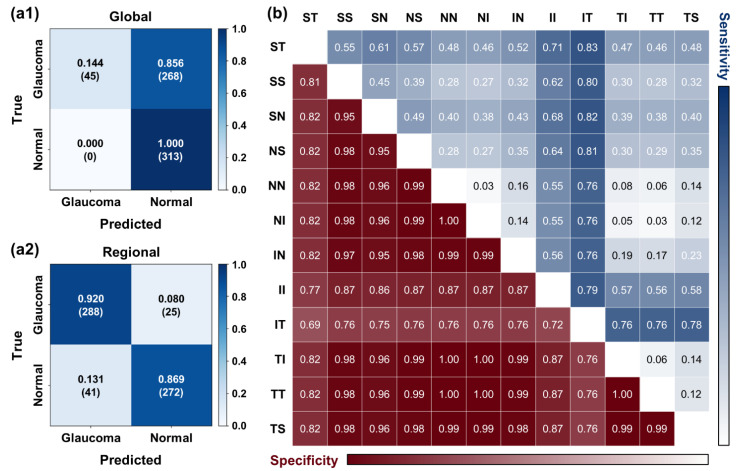
Screening performance comparison. The confusion matrix of glaucoma screening is based on the thickness level of (**a1**) the global and (**a2**) superior and inferior regions among the 12 regions. Numbers in angle bracket indicates the cases. (**b**) The sensitivity and specificity of screening results using two subsections for all possible combinations are represented as a colormap.

In Figure 6, the results of the performed screening test are represented. For the test, we composed the screening test set with 313 glaucoma cases (NTG and suspicious cases) and 313 normal cases as described in Figure 2b. The screening rule for distinguishing glaucomatous eyes simply checks the predicted categorization of averaged and regional RNFL thickness. A given eye was judged as glaucomatous by the following criteria: (1) based on global RNFL alone, a given eye is glaucomatous if the thinning level is abnormal or borderline, (2) based on regional RNFL, a given eye is glaucomatous if one of the thinning levels is abnormal or borderline and is located in superior or inferior regions (i.e., ST, SS, SN, IN, II, and IT). The results are confusion matrices as shown in Figure 6(a1,a2). Screening results based on global RNFL thickness showed 14.4% for sensitivity and 100% for specificity. Screening with regional information results showed 92% for sensitivity and 86.9% for specificity. Comparison between two confusion matrices clearly shows that screening based on the regional performs much better.

We further investigated which subregion is crucial for screening performance in terms of sensitivity and specificity. We performed the screening test using combinations of two regional subsections among 12 regions. The criteria for judging a glaucomatous eye are the same as previously. If one thinning level is abnormal or borderline, we judge a given eye as glaucoma. Figure 5b shows sensitivity and specificity for screening results from all possible combinations. The results show that screening sensitivity is relatively higher if the superior or inferior region is included. Thus, for the glaucoma patient screening, at least in the case of the normal tension glaucoma, observation of RNFL defect in the superior and inferior regions could be critical for correct diagnosis. Furthermore, when inferior-temporal (IT) subregion is included, the sensitivity of screening becomes higher than 75% while other combinations become lower than 75%. Interestingly, if these regions are included, the specificity becomes lower than 76%, which is worse than that of other combinations. This is because of the relatively wider distribution of RNFL thickness in these regions.

**Figure 7:**
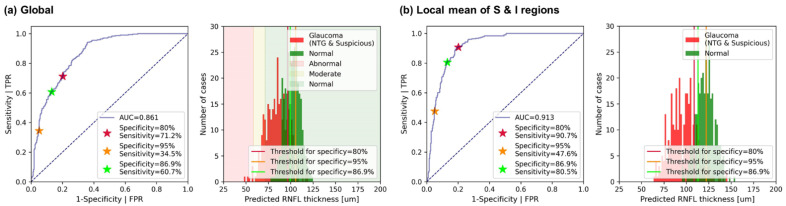
ROC analysis using (**a**) global RNFL predictions and (**b**) local mean of predicted RNFL thicknesses over superior plus inferior (ST, SS, SN, IN, II, IT) regions, which has the best score of AUC and sensitivity for specificity at 80%.

In addition, we performed an ROC analysis based on predicted global and regional RNFL thicknesses. For global RNFL thicknesses, sensitivities were 34.5% and 71.2% for specificities at 95% and 80%, respectively, as shown in Figure 7a. These values are lower than those reported in Ref. [33] (76% and 90%, respectively). We attribute this poor performance to the fact that our data was based on the normal tension glaucoma (NTG) patients. We suspect that our data distribution relative to that of Ref. [33] is closer to normal cases with thicker RNFL. For example, the mean of global RNFL thickness values from NTG patients is 84.3 μm whereas the mean of global RNFL thickness from glaucoma patients in the dataset of previous study is 68.8 μm. NTG patients show less thinning of the RNFL relative to other types of glaucoma and thus we saw thicker global value.

Furthermore, we performed ROC analysis with predictions of RNFL thickness from 12 regions and localized mean RNFL thicknesses over superior (S), nasal (N), inferior (I), temporal (T), superior plus inferior (S + I), and nasal plus temporal (N + T) regions to confirm diagnostically meaningful regions. Each local mean RNFL thickness was averaged over corresponding sections, for example, RNFL thicknesses from ST, SS, and SN sections were averaged for the local mean of the superior (S) region. Among 12 subsections, the top two highest sensitivity for specificity at 80% were confirmed as 88.2% and 70.6% from the inferior-temporal (IT) and the superior-nasal (SN) regions, respectively. The best score is given by using localized mean RNFL over S + I regions with 0.913 of AUC and 90.7% sensitivity for specificity at 80%. Sensitivity of 90.7% is higher than that based on global RNFL and comparable to the previously reported values. For a fair comparison with the screening result (Figure 6(a2)), we also report sensitivities for specificity at 86.9%. In Table 5, full results of the ROC analysis are summarized. 

## 4. Discussion

We have demonstrated the DL model-based screening protocol by inferring RNFL information from color fundus photographs. The CNN models are trained on data from two different ophthalmic imaging modalities: color fundus photography and OCT. Experimental results show that a color fundus photograph can delineate quantitative morphological changes in the retina in terms of RNFL thickness with the DL technique. These integrations of the core OCT advantages and relatively simple imaging device fundus photography by DL technique would provide low-cost, easy, and simple assessment and quantitative analysis for glaucoma screening and diagnosis. Moreover, intuitive screening information enables the classification of the normal and diseased groups with examination of the regional RNFL defect around the optic disc, which could be utilized as a new screening protocol in real world testing. 

For precise diagnosis of glaucoma, periodic observation on RNFL thinning is required because of a wide spectrum of thickness observed in normal patients [50]. Thus, it may be necessary to observe every 6 months for follow-up medical examination. However, it is very hard to perform such in-depth medical checkups in developing or underdeveloped countries with limited access to modern imaging systems such as OCT. On the other hand, qualitative examinations based on fundus photographs are limited in detecting early stage of progressive optic neuropathy due to the extensive training required. 

Recent studies suggested utilizing the DL technique that can provide effective and inexpensive glaucoma screening to a population in a limited medical environment. In these studies, they developed DL algorithms to predict global RNFL [33] or Bruch membrane opening-minimum rim width (BMO-MRW) [34] from an image of the whole optic-disc photograph. They showed predicted that quantitative structural deformation correlated with visual loss and screening capabilities based on ROC analysis. In Ref. [34], regional BMO-MRW along six different sectors was also investigated. Compared to previous studies, the proposed approach has in common that quantitative retinal neuropathy is predicted by DL technique from optic disc photographs. However, detailed localization has made for precise estimation with pairing between compartmentalized optic disc images and corresponding regional RNFL thicknesses. Screening capabilities were checked along each section and reginal combinations based on ROC analysis. From the result we have found that localized mean of RNFL thickness over superior plus inferior regions showed the best screening performance. Though ROC analysis was performed based on predicted RNFL, results demonstrate that the global RNFL thickness alone may be inadequate for accurate screening of the NTG and early-stage glaucoma patients. Results support our hypothesis that image analysis based on regional segmentation improves the outcome drastically.

The proposed simple and intuitive screening protocol showed highly sensitive performance in a single shot examination with the capability of localized RNFL estimation. The criteria of our screening protocol based on thinning level predictions could be useful to tell whether the patient’s eye is glaucomatous or normal. However, the thinning level is not sufficient to reveal how much of the RNFL defect has progressed quantitatively. In contrast, the quantitative prediction of RNFL thickness directly confirms how much retinal deformation is progressed. In a previous study, the potential of DL for predicting a progression of glaucomatous RNFL defects was investigated based on the averaged level [50]. The prediction of averaged RNFL is useful to confirm the trend of progression. However, regional-based RNFL thickness prediction can provide more detailed information. Thus, the periodic observation for checking progressive glaucoma without an OCT imaging can be an effective screening technique if localized retinal deformation can be trackable from fundus photographs.

**Figure 8:**
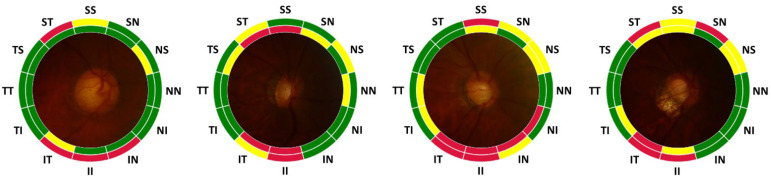
Examples of prediction failure. Model fails to estimate RNFL thickness accurately when the quality of fundus photograph is bad in terms of contrast, brightness, and blurriness and arrives at an incorrect thinning level prediction consequently. Data is based on rejected samples.

Our approach can significantly extend the limits of the qualitative and subjective information based solely on fundus photographs, but still needs further improvement before its adoption in clinical practice. We believe the accuracy of RNFL thickness prediction can be improved further if additional data is used for the training model. However, even if trained CNN can predict quantitative retinal deformation precisely, the gap between OCT-measurement and CNN based estimation critically depends on the imaging quality of the fundus photograph. In Figure 8, examples of prediction failures are represented. If the fundus photograph is not clear with blurriness, low contrast, and not enough brightness, the prediction of RNFL thickness becomes inaccurate and thus the thinning label is consequently unreliable. Thus, a clear fundus photo is an essential prerequisite for accurate prediction. In addition, the screening capability of the proposed technique would be further enhanced when the DL model includes other risk factors such as intraocular pressure, history of diabetes, hypertension and high myopia. The current approach may also include additional quantitative values such as the rim thinning, notching, and cup-to-disc ratio for the better credibility of glaucoma screening [33,34,51].

As glaucoma is known to be age-related, the global incidence is expected to rise due to the aging population. In particular, the prevalence of glaucoma is very severe in many developing countries because a large proportion of cases remain undiagnosed or sub-optimally managed due to a lack of diagnostic and screening tools. Thus, the availability of low-cost imaging devices for quick and easy screening would tremendously benefit glaucoma care in low-resource settings. For our future work, a fully developed CNN model would be tested with a low-cost, handheld fundus device integrated in an ophthalmoscope [9,10,52]. Our technique based on fundus photography and DL may be further extended to detect other retinal conditions, such as diabetic retinopathy and age-related macular degeneration while enabling accurate diagnosis even in low-resource settings.

## 5. Conclusions

We present a deep learning approach to exploit the advantages of color fundus photography for glaucoma screening. A fundus photograph is augmented with estimated RNFL thickness based on OCT data, unveiling morphological changes in the retina that cannot be obtained using color fundus photography alone. Our approach for detecting glaucoma using data from NTG patients gives a comprehensive and informative outcome, and potentially delivers a screening capability over existing methods.

## Figures and Tables

**Table 1:** Summary of dataset acquired from the NTG patients for the study.

	Collected Dataset
Number of patients	303
Number of eye examinations	557
Number of Fundus-OCT paired data Number of segmented Fundus and OCT value pairs	940 11,280
Patients’ age (years)	54.7 ± 13.9
Gender (% of female)	48.3
Intraocular pressure (mmHg)	13.87 ± 3.01
Mean deviation (dB)	−4.69 ± 5.37
Visual field index (%)	88.39 ± 15.63
Global RNFL (µm)	84.31 ± 16.21

**Table 2:** A summary of performance metrics. The mean-absolute-error (MAE), the R-squared, and the Pearson’s correlation coefficient between prediction of RNFL from trained models and true OCT-measurements.

Model	Predict	MAE	R-Squared	Pearson’s Correlation
Previous study [33]	Global RNFL	7.39 μm	0.693	0.832
Proposed CNN	Global RNFL	9.38 μm	0.502	0.710
Proposed CNN	Regional RNFL	16.22 μm	0.578	0.760

**Table 3:** The mean and standard deviation of OCT measured and trained CNN prediction on RNFL thickness (μm) in the test set along the global and regional sections.

Region	Acronym	OCT Measurement	CNN Prediction
Global	G	83.9 ± 16.1	85.0 ± 13.3
Superior temporal	ST	106.0 ± 36.3	105.3 ± 24.7
Superior	SS	107.0 ± 31.3	105.0 ± 26.9
Superior nasal	SN	101.0 ± 25.7	87.8 ± 20.8
Nasal superior	NS	78.7 ± 21.1	76.9 ± 16.9
Nasal	NN	57.6 ± 12.7	61.2 ± 11.0
Nasal inferior	NI	65.4 ± 16.7	71.3 ± 13.1
Inferior nasal	IN	93.9 ± 24.4	91.7 ± 18.4
Inferior	II	104.1 ± 38.2	101.4 ± 26.2
Inferior temporal	IT	87.2 ± 41.2	88.3 ± 22.5
Temporal inferior	TI	64.2 ± 16.8	71.8 ± 11.6
Temporal	TT	61.6 ± 13.4	70.2 ± 11.4
Temporal superior	TS	79.9 ± 22.2	79.7 ± 15.4

**Table 4:** Summary of estimated RNFL (μm) from glaucoma, suspicious, and normal groups for global and 12 regions.

	G	ST	SS	SN	NS	NN	NI	IN	II	IT	TI	TT	TS
Glaucoma	85.0	105.2	104.9	87.7	76.9	61.2	71.2	91.7	101.3	88.2	71.8	70.1	79.7
	±13.3	±24.8	±27.0	±20.8	±16.9	±10.9	±13.2	±18.4	±26.2	±22.5	±11.6	±11.4	±15.4
Suspicious	86.5	100.8	108.7	94.8	83.9	70.6	80.7	94.8	107.7	90.4	72.2	71.5	78.5
	±10.2	±20.3	±17.1	±15.5	±13.5	±10.6	±12.7	±15.6	±19.9	±18.1	±8.4	±8.2	±9.9
Normal	104.1	133.2	128.2	114.8	99.4	78.7	90.1	115.5	133.6	124.9	81.8	80.2	91.5
	±7.5	±17.8	±16.8	±15.9	±15.4	±12.4	±14.5	±14.8	±17.2	±16.8	±8.7	±8.3	±11.2

**Table 5:** Summary of ROC analysis based on section-wise predicted RNFL thicknesses and localized mean over superior (S), nasal (N) inferior (I), temporal (T), superior plus inferior (S + I), and nasal plus temporal (N + T). * Refer to the highest sensitivity and ** to the second highest sensitivity for specificity at 80% among 12 subsections. † Refer to the best scored overall results.

Section	Area Under Curve	Sensitivity for Specificity at 95%	Sensitivity for Specificity at 80%	Sensitivity for Specificity at 86.9%	Threshold RNFL for Specificity at 95%	Threshold RNFL for Specificity at 80%	Threshold RNFL for Specificity at 86.9%
Global	0.861	34.5%	71.3	60.7	105.57	96.73	99.41
ST	0.779	17.6%	53.7%	41.2%	144.11	123.65	130.56
SS	0.765	8.6%	56.2%	37.1%	149.43	121.19	131.83
SN **	0.835	24.0%	70.6%	50.5%	125.85	105.22	112.96
NS	0.816	24.6%	62.9%	48.6%	108.95	92.12	97.58
NN	0.807	18.2%	63.3%	43.1%	87.23	72.65	77.27
NI	0.815	23.3%	66.1%	50.5%	100.66	84.20	89.56
IN	0.826	30.4%	70.0%	59.4%	122.32	106.19	110.97
II	0.830	19.8%	61.0%	40.3%	144.03	125.10	133.11
IT *	0.848	21.1%	88.2%	60.4%	129.52	101.24	112.08
TI	0.725	16.3%	49.2%	35.5%	89.20	79.97	83.57
TT	0.713	16.3%	43.8%	34.2%	86.08	79.60	81.77
TS	0.731	10.9%	49.2%	31.0%	103.68	88.41	93.26
S	0.862	29.7%	77.0%	60.7%	128.93	113.14	118.34
N	0.854	33.6%	73.5%	56.6%	92.94	81.79	86.37
I	0.899	36.4%	85.6%	71.9%	124.22	108.37	113.79
T	0.765	18.9%	54.3%	43.8%	89.59	81.23	83.60
S + I ^†^	0.913	47.6%	90.7%	80.5%	122.07	108.73	113.02
N + T	0.868	32.6%	76.7%	60.7%	88.86	80.00	83.04

## Data Availability

The data presented in this study are available on request from the corresponding author.

## References

[1] Weinreb R.N., Khaw P.T. (2004). Primary open-angle glaucoma. Lancet.

[2] Greco A., Rizzo M.I., De Virgilio A., Gallo A., Fusconi M., de Vincentiis M. (2016). Emerging Concepts in Glaucoma and Review of the Literature. Am. J. Med..

[3] Killer H.E., Pircher A. (2018). Normal tension glaucoma: Review of current understanding and mechanisms of the pathogenesis. Eye.

[4] Haleem M.S., Han L., van Hemert J., Li B. (2013). Automatic extraction of retinal features from colour retinal images for glaucoma diagnosis: A review. Comput. Med. Imaging Graph..

[5] Myers J.S., Fudemberg S.J., Lee D. (2018). Evolution of optic nerve photography for glaucoma screening: A review. Clin. Exp. Ophthalmol..

[6] Bock R., Meier J., Nyúl L.G., Hornegger J., Michelson G. (2010). Glaucoma risk index:Automated glaucoma detection from color fundus images. Med. Image Anal..

[7] Badalà F., Nouri-Mahdavi K., Raoof D.A., Leeprechanon N., Law S.K., Caprioli J. (2007). Optic Disk and Nerve Fiber Layer Imaging to Detect Glaucoma. Am. J. Ophthalmol..

[8] Niemeijer M., Ginneken B.v., Cree M.J., Mizutani A., Quellec G., Sanchez C.I., Zhang B., Hornero R., Lamard M., Muramatsu C. (2010). Retinopathy Online Challenge: Automatic Detection of Microaneurysms in Digital Color Fundus Photographs. IEEE Trans. Med. Imaging.

[9] Jin K., Lu H., Su Z., Cheng C., Ye J., Qian D. (2017). Telemedicine screening of retinal diseases with a handheld portable non-mydriatic fundus camera. BMC Ophthalmol..

[10] Upadhyaya S., Agarwal A., Rengaraj V., Srinivasan K., Newman Casey P.A., Schehlein E. (2021). Validation of a portable, non-mydriatic fundus camera compared to gold standard dilated fundus examination using slit lamp biomicroscopy for assessing the optic disc for glaucoma. Eye.

[11] Reis A.S.C., O’Leary N., Yang H., Sharpe G.P., Nicolela M.T., Burgoyne C.F., Chauhan B.C. (2012). Influence of Clinically Invisible, but Optical Coherence Tomography Detected, Optic Disc Margin Anatomy on Neuroretinal Rim Evaluation. Investig. Ophthalmol. Vis. Sci..

[12] Adhi M., Duker J.S. (2013). Optical coherence tomography--current and future applications. Curr. Opin. Ophthalmol..

[13] Thomas D., Duguid G. (2004). Optical coherence tomography—A review of the principles and contemporary uses in retinal investigation. Eye.

[14] Wu H., De Boer J.F., Chen T.C. (2012). Diagnostic capability of spectral-domain optical coherence tomography for glaucoma. Am. J. Ophthalmol..

[15] Povazay B., Hofer B., Hermann B.M., Unterhuber A., Morgan J.E., Glittenberg C., Binder S., Drexler W. (2007). Minimum distance mapping using three-dimensional optical coherence tomography for glaucoma diagnosis. J. Biomed. Opt..

[16] Bussel I.I., Wollstein G., Schuman J.S. (2014). OCT for glaucoma diagnosis, screening and detection of glaucoma progression. Br. J. Ophthalmol..

[17] Chen T.C. (2009). Spectral domain optical coherence tomography in glaucoma: Qualitative and quantitative analysis of the optic nerve head and retinal nerve fiber layer (an AOS thesis). Trans. Am. Ophthalmol. Soc..

[18] Moreno P.A.M., Konno B., Lima V.C., Castro D.P.E., Castro L.C., Leite M.T., Pacheco M.A.M.M., Lee J.M., Prata T.S. (2011). Spectral-domain optical coherence tomography for early glaucoma assessment: Analysis of macular ganglion cell complex versus peripapillary retinal nerve fiber layer. Can. J. Ophthalmol..

[19] Sakata K., Sakata L.M., Sakata V.M., Santini C., Hopker L.M., Bernardes R., Yabumoto C., Moreira A.T. (2007). Prevalence of glaucoma in a South brazilian population: Projeto Glaucoma. Investig. Ophthalmol. Vis. Sci..

[20] Takahashi H., Tampo H., Arai Y., Inoue Y., Kawashima H. (2017). Applying artificial intelligence to disease staging: Deep learning for improved staging of diabetic retinopathy. PLoS ONE.

[21] Sahlsten J., Jaskari J., Kivinen J., Turunen L., Jaanio E., Hietala K., Kaski K. (2019). Deep learning fundus image analysis for diabetic retinopathy and macular edema grading. Sci. Rep..

[22] Gulshan V., Peng L., Coram M., Stumpe M.C., Wu D., Narayanaswamy A., Venugopalan S., Widner K., Madams T., Cuadros J. (2016). Development and validation of a deep learning algorithm for detection of diabetic retinopathy in retinal fundus photographs. JAMA.

[23] Ting D.S.W., Cheung C.Y.-L., Lim G., Tan G.S.W., Quang N.D., Gan A., Hamzah H., Garcia-Franco R., San Yeo I.Y., Lee S.Y. (2017). Development and validation of a deep learning system for diabetic retinopathy and related eye diseases using retinal images from multiethnic populations with diabetes. JAMA.

[24] Raju M., Pagidimarri V., Barreto R., Kadam A., Kasivajjala V., Aswath A. (2017). Development of a deep learning algorithm for automatic diagnosis of diabetic retinopathy. MEDINFO 2017: Precision Healthcare through Informatics.

[25] Li Z., He Y., Keel S., Meng W., Chang R.T., He M. (2018). Efficacy of a deep learning system for detecting glaucomatous optic neuropathy based on color fundus photographs. Ophthalmology.

[26] Sengupta S., Singh A., Leopold H.A., Gulati T., Lakshminarayanan V. (2020). Ophthalmic diagnosis using deep learning with fundus images–a critical review. Artif. Intell. Med..

[27] Christopher M., Hoseini P., Walker E., Proudfoot J.A., Bowd C., Fazio M.A., Girkin C.A., De Moraes C.G., Liebmann J.M., Weinreb R.N. (2022). A deep learning approach to improve retinal structural predictions and aid glaucoma neuroprotective clinical trial design. Ophthalmol. Glaucoma.

[28] Ali R., Hardie R.C., Narayanan B.N., Kebede T.M. (2022). IMNets: Deep Learning Using an Incremental Modular Network Synthesis Approach for Medical Imaging Applications. Appl. Sci..

[29] Jammal A.A., Thompson A.C., Mariottoni E.B., Berchuck S.I., Urata C.N., Estrela T., Wakil S.M., Costa V.P., Medeiros F.A. (2020). Human Versus Machine: Comparing a Deep Learning Algorithm to Human Gradings for Detecting Glaucoma on Fundus Photographs. Am. J. Ophthalmol..

[30] Jampel H.D., Friedman D., Quigley H., Vitale S., Miller R., Knezevich F., Ding Y. (2009). Agreement among glaucoma specialists in assessing progressive disc changes from photographs in open-angle glaucoma patients. Am. J. Ophthalmol..

[31] Varma R., Steinmann W.C., Scott I.U. (1992). Expert agreement in evaluating the optic disc for glaucoma. Ophthalmology.

[32] Tielsch J.M., Katz J., Quigley H.A., Miller N.R., Sommer A. (1988). Intraobserver and interobserver agreement in measurement of optic disc characteristics. Ophthalmology.

[33] Medeiros F.A., Jammal A.A., Thompson A.C. (2019). From machine to machine: An OCT-trained deep learning algorithm for objective quantification of glaucomatous damage in fundus photographs. Ophthalmology.

[34] Thompson A.C., Jammal A.A., Medeiros F.A. (2019). A deep learning algorithm to quantify neuroretinal rim loss from optic disc photographs. Am. J. Ophthalmol..

[35] Zhao J., Solano M.M., Oldenburg C.E., Liu T., Wang Y., Wang N., Lin S.C. (2019). Prevalence of normal-tension glaucoma in the Chinese population: A systematic review and meta-analysis. Am. J. Ophthalmol..

[36] Kim M., Kim T.-W., Park K.H., Kim J.M. (2012). Risk factors for primary open-angle glaucoma in South Korea: The Namil study. Jpn. J. Ophthalmol..

[37] Shields M.B. (2008). Normal-tension glaucoma: Is it different from primary open-angle glaucoma?. Curr. Opin. Ophthalmol..

[38] Woo S., Park K.H., Kim D. (2003). Comparison of localised nerve fibre layer defects in normal tension glaucoma and primary open angle glaucoma. Br. J. Ophthalmol..

[39] Thonginnetra O., Greenstein V.C., Chu D., Liebmann J.M., Ritch R., Hood D.C. (2010). Normal versus high tension glaucoma: A comparison of functional and structural defects. J. Glaucoma.

[40] Suh M., Kim D., Kim Y., Kim T., Park K. (2010). Patterns of progression of localized retinal nerve fibre layer defect on red-free fundus photographs in normal-tension glaucoma. Eye.

[41] LeCun Y., Bengio Y. (1995). Convolutional networks for images, speech, and time series. Handb. Brain Theory Neural Netw..

[42] Ioffe S., Szegedy C. Batch normalization: Accelerating deep network training by reducing internal covariate shift. In Proceedings of International Conference on Machine Learning.

[43] Nair V., Hinton G.E. Rectified linear units improve restricted boltzmann machines. In Proceedings of ICML.

[44] Kingma D.P., Ba J. (2014). Adam: A method for stochastic optimization. arXiv.

[45] Bendschneider D., Tornow R.P., Horn F.K., Laemmer R., Roessler C.W., Juenemann A.G., Kruse F.E., Mardin C.Y. (2010). Retinal nerve fiber layer thickness in normals measured by spectral domain OCT. J. Glaucoma.

[46] Chaglasian M., Fingeret M., Davey P.G., Huang W.-C., Leung D., Ng E., Reisman C.A. (2018). The development of a reference database with the Topcon 3D OCT-1 Maestro. Clin. Ophthalmol. (Auckl. NZ).

[47] Shin J.W., Sung K.R., Song M.K. (2020). Ganglion cell-inner plexiform layer and retinal nerve fiber layer changes in glaucoma suspects enable prediction of glaucoma development. Am. J. Ophthalmol..

[48] Hwang Y.H., Kim Y., Chung J.K., Lee K.B. (2018). Glaucomatous progression in the retinal nerve fibre and retinal ganglion cell-inner plexiform layers determined using optical coherence tomography-guided progression analysis. Clin. Exp. Optom..

[49] Lee W.J., Kim Y.K., Park K.H., Jeoung J.W. (2017). Trend-based analysis of ganglion cell–inner plexiform layer thickness changes on optical coherence tomography in glaucoma progression. Ophthalmology.

[50] Medeiros F.A., Jammal A.A., Mariottoni E.B. (2021). Detection of progressive glaucomatous optic nerve damage on fundus photographs with deep learning. Ophthalmology.

[51] Phene S., Dunn R.C., Hammel N., Liu Y., Krause J., Kitade N., Schaekermann M., Sayres R., Wu D.J., Bora A. (2019). Deep learning and glaucoma specialists: The relative importance of optic disc features to predict glaucoma referral in fundus photographs. Ophthalmology.

[52] Prince J., Thompson A., Mwanza J.-C., Tolleson-Rinehart S., Budenz D.L. (2022). Glaucoma Screening Using an iPad-Based Visual Field Test in a West African Population. Ophthalmol. Glaucoma.

